# Sulfoxaflor Impairs Bumblebee Memory Across Moderate and Extreme Temperatures

**DOI:** 10.1002/ece3.72073

**Published:** 2025-09-05

**Authors:** Rinoa Hicks, Szymon Szymański, Elena Couper Coombs, Harry Siviter

**Affiliations:** ^1^ School of Biological Sciences University of Bristol Bristol UK

**Keywords:** climate change, cognition, insecticide, pesticide, pollinators

## Abstract

Pesticide exposure and climate change are key drivers of pollinator declines. Exposure to certain pesticides and high temperatures can influence the cognitive ability of insect pollinators, such as bees, but little is known about how these stressors interact. As central place foragers, bees must learn and remember floral cues, and so impaired memory may influence foraging efficiency and fitness. While individual exposure to specific pesticides and high temperatures can impair bee memory, the interactions between these two key stressors remain poorly understood. Here, using a free‐moving proboscis extension reflex experimental design, we assessed the impact of an acute, field‐realistic dose of the insecticide sulfoxaflor on bumblebee ( *Bombus terrestris* ) memory across a range of environmentally relevant temperatures (12°C–36°C). We found that exposure to sulfoxaflor impaired bumblebee memory, despite the study using a pesticide dose that could be experienced during a single foraging event, rather than a prolonged chronic exposure regime. In contrast to previous findings, we found no impact of temperature exposure on bumblebee memory and no significant interaction between sulfoxaflor exposure and temperature. Our results, alongside previous research, demonstrate that sulfoxaflor can have negative sub‐lethal impacts on important pollinators. Legislation that either (i) restricts the outdoor use of sulfoxaflor entirely, as seen in the EU, or (ii) limits use to non‐flowering crops, will benefit pollinators, and limit the unintended consequences of intensive agriculture.

## Graphical abstract

Bumblebees rely on their memory to navigate a complicated landscape and floral marketplace. When foraging, bees are often exposed to a multitude of anthropogenic stressors, but how these stressors influence bee cognition, both individually and in combination, remains poorly understood. We found, in a fully crossed experiment, that the insecticide sulfoxaflor, but not extreme temperatures, impaired bumblebee memory, adding to a growing body of evidence indicating that this insecticide can have negative impacts on important insect pollinators.

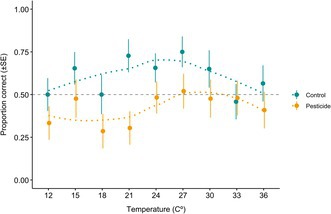

## 1 Introduction

An estimated 90% of angiosperms and 35% of agricultural crops are pollinator dependent, with widely documented declines in pollinating insects threatening both food production and ecosystem stability (Janousek et al. 2023; Klein et al. 2007; Ollerton et al. 2011; Tong et al. 2023; Zattara and Aizen 2021). Loss of habitat, intensive agriculture, pesticide exposure, climate change, as well as parasites and pathogens are all key drivers of these declines (Kerr et al. 2015; Nicholson et al. 2024; Ravoet et al. 2014; Rundlöf et al. 2015; Vray et al. 2019). While individually these stressors can be detrimental to pollinators, they may also interact and result in synergistic negative interactions, whereby the effect of the combined stressors is greater than the sum of their combined individual effects (Siviter, Johnson, and Muth 2021). Consequently, quantifying the interactions between highly prevalent anthropogenic stressors on insect pollinators is vital for understanding their true impact (Vanbergen and The Insect Pollinators Initiative 2013).

Exposure to pesticides and climate change are two of the key drivers of the declines in pollinating insects (Kazenel et al. 2024; Nicholson et al. 2024; Rundlöf et al. 2015). Most insecticides are neurotoxins and may have sub‐lethal effects on pollinator behaviour and physiology (Crall et al. 2018; Kenna et al. 2019; Siviter, Johnson, and Muth 2021), which can have downstream consequences for populations (Nicholson et al. 2024; Rundlöf et al. 2015; Whitehorn et al. 2012). Likewise, extreme temperature events, which are increasing in response to human‐induced climate change, can have both lethal and sub‐lethal impacts on pollinators (Gérard et al. 2023; Kazenel et al. 2024; Martinet et al. 2021). As climate change intensifies, extreme temperature events will become more frequent, and pesticide use will subsequently increase as farmers need more treatments to protect weather‐damaged crops (Delcour et al. 2015; Noyes et al. 2009). Consequently, pollinators will be increasingly exposed to extreme temperatures and pesticides simultaneously (European Food Safety Authority (EFSA) et al. 2019; Janousek et al. 2023; Mitchell et al. 2017). Indeed, a horizon scanning exercise conducted by 72 pollination experts identified (i) novel pesticides and (ii) extreme climate events as two of the most significant emerging threats to insect pollinators and their pollination services (Brown et al. 2016).

Individually, exposure to pesticides and extreme heat can influence the foraging behaviour of pollinating insects, but little is known about how they interact (Kenna et al. 2023). Nesting pollinators are central place foragers and navigate a dynamic floral landscape (Sommer et al. 2022; Woodgate et al. 2016). They must learn and remember the locations and floral cues associated with floral rewards to forage efficiently (Bertrand et al. 2021; Saleh et al. 2007; Schiestl and Johnson 2013). For social bees, an inability to forage effectively as a consequence of impaired memory may have downstream consequences for colony resource intake (Raine and Chittka 2008)—a key determinant of reproductive output (Gegear et al. 2021; Raine and Chittka 2008; Vaudo et al. 2018). Moreover, a failure to learn their environment can result in individual bees being unable to return to their colony, which can lead to direct mortality (Eckel et al. 2023). Exposure to certain pesticides, such as neonicotinoids, can influence pollinator learning, memory and navigation (Samuelson et al. 2016; Siviter, Koricheva, et al. 2018; Stanley et al. 2015). Less is known about the impact of temperature on bumblebee learning and memory, but exposure to extreme heat (32°C for 2 h 40 min) can influence learning and memory (Gérard, Amiri, et al. 2022). Similarly, exposure to higher temperatures (40°C for 2 h 45 min) can impair bumblebee antennal responses to floral scents (Nooten et al. 2024). To our knowledge, no studies have assessed the impact of temperature and pesticide exposure on bumblebee learning or memory, but exposure to pesticides and heightened temperatures can also result in synergistic negative impacts on solitary bee ( *Osmia cornuta* ) longevity and behaviour (Albacete et al. 2023), as well as bumblebee flight distance (Kenna et al. 2023).

**FIGURE 1:**
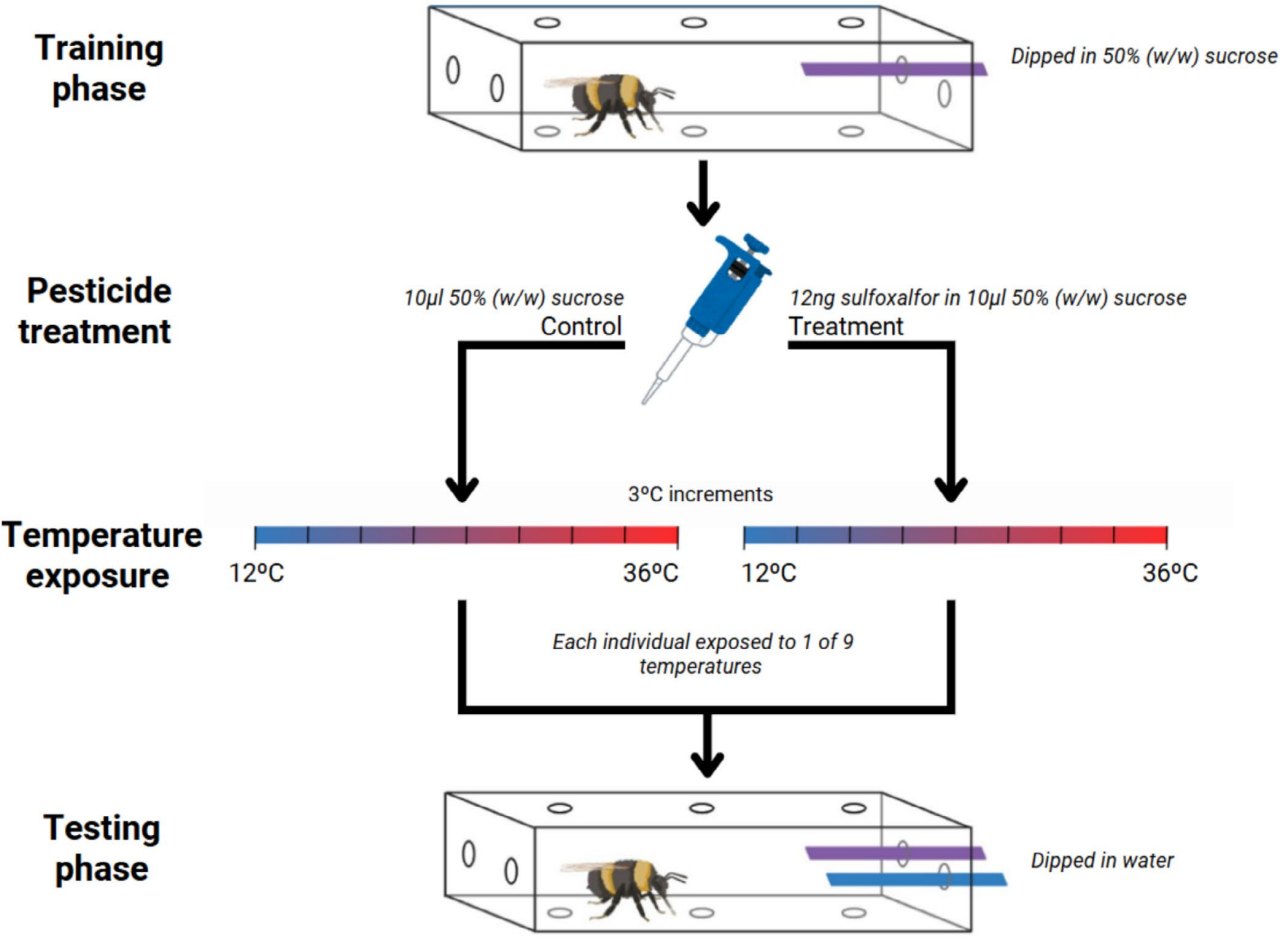
Schematic visualising the apparatus and workflow of the experiment. Bees were trained to associate a coloured stimulus (CS+) with a sucrose reward via absolute conditioning. A blue or purple strip of card (colour counterbalanced between individuals) was dipped into 50% (w/w) sucrose and presented to the bee, which was allowed to feed for 3 s. This process was repeated for five trials with an intertrial interval of 10 min. Post training, the bees were exposed to a predetermined pesticide and temperature exposure regime. Bees were fed a 10 μL droplet of a 50% (w/w) sucrose solution which was either pesticide free (control) or a droplet containing an acute dose of sulfoxaflor (12 ng). They were then subsequently exposed to one of a range of temperatures (between 12°C and 36°C) for one hour. Memory was subsequently tested by presenting both coloured cards dipped in water and recording which stimulus was touched first.

Here, we assessed the impact of an acute dose of the insecticide sulfoxaflor on bumblebee ( *Bombus terrestris* ) memory across a range of ecologically relevant temperatures (12°C–36°C). Sulfoxaflor is a neurotoxin that acts as an agonist of nicotinic acetylcholine receptors (NAChRs) (Sparks et al. 2013). It has been banned for outdoor agricultural use in the European Union but is still available globally. While sulfoxaflor can impair bumblebee fitness (Linguadoca et al. 2021; Siviter, Koricheva, et al. 2018; Siviter et al. 2019) and behaviour at field‐realistic concentrations (Kenna et al. 2023; Tamburini et al. 2021), there is no evidence of this insecticide impairing bumblebee memory (Siviter et al. 2019; Straw et al. 2023; Vaughan et al. 2022). Here, using a free‐moving proboscis extension reflex experimental design (Muth et al. 2018), we conditioned bumblebees to learn a colour association before exposing them to a temperature and pesticide combination in a fully crossed experimental design (Figure 1). On the basis of previous research (Siviter et al. 2019; Vaughan et al. 2022), we predicted that sulfoxaflor would not impair bee memory at moderate temperatures (e.g., 21°C–27°C) (Couvillon et al. 2010; Vogt 1986) but hypothesised that the pesticide would synergistically interact with both at high and low and temperature extremes to impair bumblebee memory.

## 2 Methodology

### 2.1 Colony Husbandry and Training Protocol

We purchased nine commercial bumblebee ( *Bombus terrestris audax* ) colonies (AgroBio, Spain) containing approximately 50–60 workers and maintained them under laboratory conditions (26°C, 60% relative humidity). The colonies were provided with *ad libitum* sucrose solution (30% w/w) and 3 teaspoons of pollen (AgroBio) every 2–3 days (Bridges et al. 2023; Siviter and Muth 2022). We provided bees with 30% sucrose (w/w) (rather than 50% w/w) to increase motivation for conditioning trials (see below for further details).

Individual workers were extracted from a colony and placed into transparent plastic tubes (3 × 3 × 15 cm) (Figure 1). The bees were then left in the tubes at 26°C for 1 h to habituate prior to the training phase. Bees (*n* = 475) were trained to associate a coloured stimulus with a sucrose reward (CS+) via absolute conditioning. We dipped either a blue or purple strip of card into 50% (w/w) sucrose solution and used it to stimulate the antenna of each subject (Figure 1) (Gray et al. 2024; Muth et al. 2018; Siviter and Muth 2022). Once a bee had extended its proboscis, the worker was given 3 s to feed before the stimulus was removed. We repeated this process for a total of five trials with an intertrial interval of 10 min (Bitterman et al. 1983). The colour of the CS+ was counterbalanced between bees (i.e., half of bees were trained to blue and half to purple), and the position of the CS+ was alternated randomly between the left and right tube openings (Muth et al. 2018). Bees that did not extend their proboscis in response to the CS+ were omitted from the experiment (control *n* = 13, treatment *n* = 8). Once training was complete, the bees were exposed to a predetermined combination of treatment (control or pesticide) and temperature.

### 2.2 Pesticide and Temperature Exposure

We exposed bees to an acute, field‐realistic dose of sulfoxaflor across a gradient of temperatures. The fully crossed experimental design consisted of 18 experimental groups (Figure 1); 9 groups (control) were fed 10 μL of untreated 50% (w/w) sucrose solution, while the other 9 groups (treatment) consumed 12 ng of sulfoxaflor in 10 μL of 50% (w/w) sucrose solution. This dose reflects a worst‐case scenario, where a bee feeds on a crop sprayed while in bloom (European Food Safety Authority (EFSA) et al. 2019). Sulfoxaflor is banned for outdoor use in the in the EU, but it is still used globally where it can be permitted on bee‐attractive plants whilst they are flowering. For example, New Zealand permits the use of sulfoxaflor on flowering vegetables (Corteva Agriscience New Zealand, n.d.); in South Africa, it can be applied on a range of flowering fruits and vegetables during bloom (Corteva Agriscience South Africa, n.d.); Australia permits sulfoxaflor use on canola (Australian Pesticides and Veterinary Medicines Authority 2013; Corteva Agriscience Australia, n.d.); and in the US, sulfoxaflor can be used on bee‐visited crops (e.g., citrus crops) once during bloom (EPA 2019). Thus, the sulfoxaflor dose used in this experiment is field‐realistic in several jurisdictions but not others. Furthermore, the utilised dose is still considered conservative, as sulfoxaflor concentrations in the nectar of treated crops can reach doses that can have lethal consequences on foraging bees (European Food Safety Authority (EFSA) et al. 2019; Linguadoca et al. 2021). The dose is higher than the amount used in Siviter et al. (2019) and the same as the amount used by Vaughan et al. (2022) on bumblebees. Neither study found any evidence of an adverse effect of sulfoxaflor on bumblebee learning and memory (Siviter et al. 2019; Vaughan et al. 2022).

We created the stock solution of sulfoxaflor by diluting powdered sulfoxaflor (10 mg; Stratech Scientific Ltd., Ely, UK) with distilled water, which was then added to a 50% (w/w) sucrose solution. This was stored at −20°C until needed for both control and pesticide groups; 10 μL of the relevant sucrose solution (see above) was pipetted into the plastic tubes for the bees to consume. Bees that did not consume the sucrose solution were assumed to be unmotivated and removed from the experiment (control *n* = 2, treatment *n* = 2). 15 min after the final bee had ingested the given solution, the bees were placed into an incubator for 1 h under their respective temperature exposures (Figure 1).

Bees were exposed to one of nine temperatures ranging from 12°C to 36°C in 3°C increments (Figure 1) for 1 h. We intended to replicate exposure during foraging, since in the field, *B. terrestris* typically nest underground, where temperature variations are likely less severe than in ambient air (Gradišek et al. 2023), and colonies are able to actively thermoregulate (Vogt 1986). The temperature range used reflects a range of temperatures at which bumblebees are known to forage (Kenna et al. 2021; Kwon and Saeed 2003), including hotter temperatures which are less optimal for cold‐adapted bumblebees (Hemberger et al. 2023). We considered moderate temperatures to be between 21°C and 27°C as these are the temperatures at which bumblebee foraging activity is highest (Couvillon et al. 2010) and colony engagement in thermoregulatory behaviour is least (Vogt 1986). Subjects were exposed to the temperature for 1 h for optimal pesticide absorption and to reflect a typical foraging duration (Samuelson et al. 2016; Westphal et al. 2006).

### 2.3 Testing Protocol

Once the temperature exposure period was completed, the bees were moved back into the testing room (26°C) for 10 min for recovery and acclimatisation before the memory test. In this trial, we presented the bees with both the blue and purple strips of card dipped in water (Figure 1). Following the Free‐moving Proboscis Extension Reflex protocol (FMPER) (Muth et al. 2018), bees were left to approach the stimuli, and we recorded the first choice. A choice was defined as the bee making first contact with the card using either its antennae or proboscis (Muth et al. 2018). Both stimuli were removed when the choice was made. The experimenters trained, treated and tested between 7 and 11 bees in each batch. All bees in a single batch were placed into the same temperature treatment, while the CS+ colour (blue or purple) and the pesticide treatment (sulfoxaflor or control) were split equally between bees in each batch. Once all the subjects were tested, the bees were frozen and their intertegular distance was measured with electronic callipers (Cane 1987). Our final sample size was 430 bees (control 12°C *n* = 28, 15°C *n* = 26, 18°C *n* = 20, 21°C *n* = 22, 24°C *n* = 32, 27°C *n* = 24, 30°C *n* = 20, 33°C *n* = 24, 36°C *n* = 23; pesticide 12°C *n* = 24, 15°C *n* = 21, 18°C *n* = 21, 21°C *n* = 23, 24°C *n* = 29, 27°C *n* = 25, 30°C *n* = 21, 33°C *n* = 25, 36°C *n* = 22). The experimenters (RH & ECC) were blind to pesticide treatment but not the temperature group.

### 2.4 Statistical Analysis

We used an information‐theoretic model selection approach. We created a full model with all measured factors (temperature, pesticide, bee size and CS+ colour). The full model and all possible subset models were compared using model selection. Model selection was performed based on Akaike Information Criterion corrected for small sample sizes (AICc). To assess bumblebee memory, we analysed the accuracy of their response to the coloured stimuli. Correct responses were coded as ‘1’, and incorrect responses were coded as ‘0’. Generalised linear mixed‐effect models (GLMM) with binomial distributions were used to analyse the data. The full model included treatment (pesticide or control), temperature (12°C–36°C), bee size (intertegular distance), CS+ colour (blue or purple) and the interaction between pesticide and temperature. Colony was specified as a random factor. Multiple models had an ΔAICc ≤ 2 so we used model averaging to produce parameter estimates and 95% confidence intervals (Burnham and Anderson 2004). A list of all selected models and outputs can be found in Table S1 and Table S2. The analysis was conducted in R (version 4.4.1) using the packages *MuMin*, *ggplot2, ggpubr and lme4* (Bartoń 2024; Bates et al. 2015; Kassambara 2016; Wickham 2016).

## 3 Results

**FIGURE 2:**
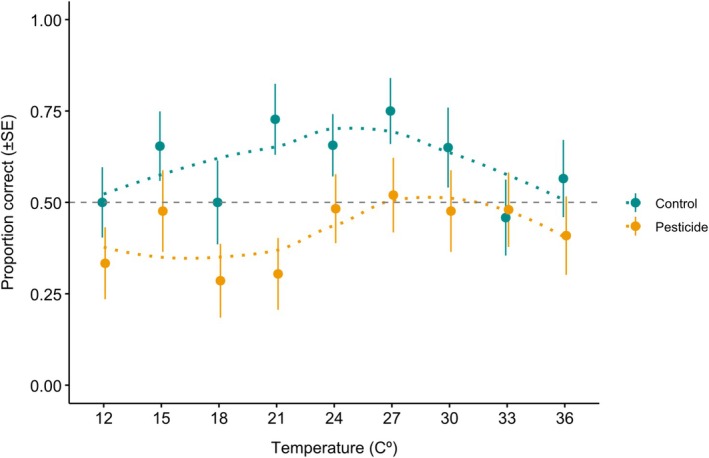
Proportion of correct responses (±SE) at different temperatures and treatments. No evidence of a temperature‐sensitive effect was found, but sulfoxaflor exposure impaired memory. Note that the points are horizontally offset between the temperatures to avoid overlapping and improve clarity. (Control: 12°C *n* = 28, 15°C *n* = 26, 18°C *n* = 20, 21°C *n* = 22, 24°C *n* = 32, 27°C *n* = 24, 30°C *n* = 20, 33°C *n* = 24, 36°C *n* = 23: Treatment: 12°C *n* = 24, 15°C *n* = 21, 18°C *n* = 21, 21°C *n* = 23, 24°C *n* = 29, 27°C *n* = 25, 30°C *n* = 21, 33°C *n* = 25, 36°C *n* = 22).

**FIGURE 3:**
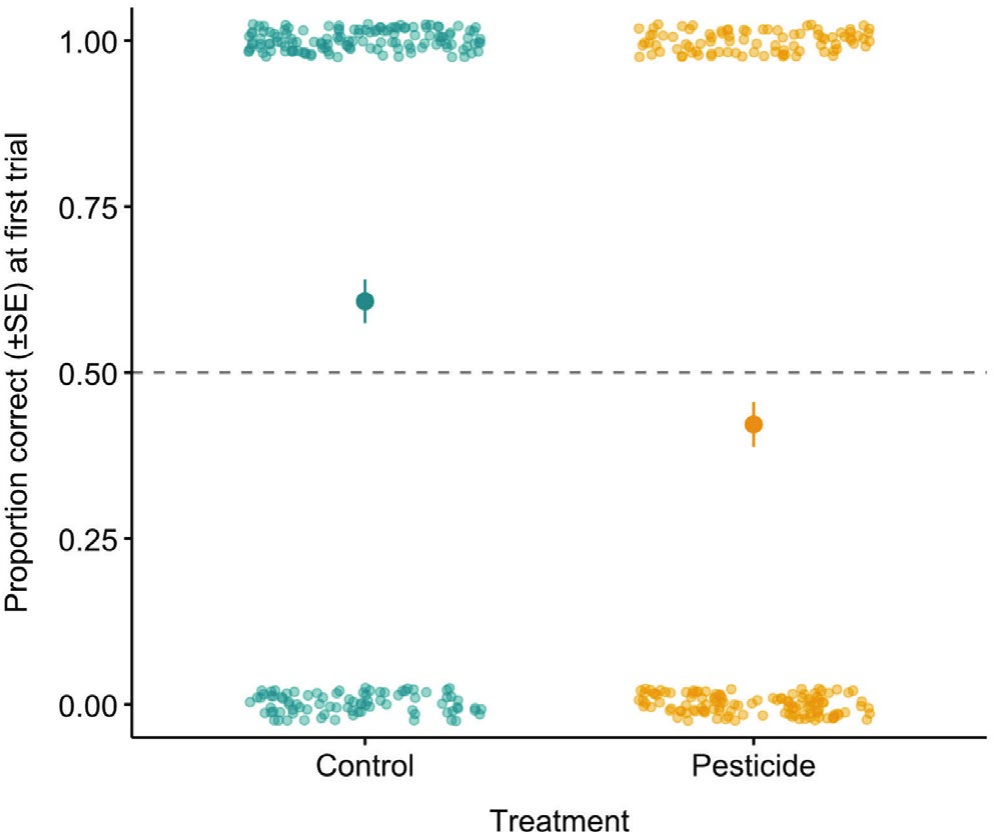
Proportion of correct responses (±SE) for bees in the control and pesticide groups, across all temperatures. Responses were coded ‘1’ for a correct choice and ‘0’ for an incorrect choice – data points at these values indicate the results of individual bees (Control *n* = 219, Pesticide *n* = 211).

Exposure to an acute dose of sulfoxaflor impaired bumblebee memory (Figure 2, treatment: parameter estimate (PE) = −0.74, 95% confidence interval (CI) = −1.14 to −0.34). We found no statistical evidence to suggest that temperature exposure influences bumblebee memory (Figure 2, temperature: PE = 0.011, CI = −0.013 to 0.018). We also found no evidence of an interaction effect between pesticide and temperature (temperature*treatment: AICc ≥ 2). Independent of temperature, bumblebees that consumed sulfoxaflor responded with a correct decision in 42% of trials, compared to 61% in the control group (Figure 3). We found no evidence to suggest that bee size or CS+ colour influenced bumblebee memory (Table S1 and Table S2).

## 4 Discussion

We found that exposure to sulfoxaflor, at a worst‐case but field‐realistic dose, significantly impaired bumblebee memory. We found no statistical evidence of a temperature‐sensitive effect on bumblebee memory and no evidence of an interaction between pesticide exposure and temperature. Our results are the first to demonstrate a negative impact of sulfoxaflor on bumblebee memory at field‐realistic concentrations (Siviter et al. 2019; Vaughan et al. 2022) and demonstrate further that this insecticide can have significant sub‐lethal effects on pollinating insects at field‐realistic levels (Boff et al. 2021; Cartereau et al. 2022; Kenna et al. 2023; Linguadoca et al. 2021; Siviter, Koricheva, et al. 2018; Tamburini et al. 2021).

Bumblebees exposed to sulfoxaflor, regardless of the temperature, were less likely to make a ‘correct’ choice in a free moving experimental protocol—confirming that this pesticide can impair bumblebee memory and cognitive function. Sulfoxaflor is a neurotoxin and has a similar mode of action as neonicotinoids, acting as an agonist of nicotinic acetylcholine receptors (NAChRs) (Sparks et al. 2013). Neonicotinoids, which have also been shown to impair bee cognition at field realistic levels (Siviter, Richman, and Muth 2021), are known to induce alterations in neuron organelles, decrease the density of synaptic boutons in the mushroom bodies and increase neuron death (Cabirol and Haase 2019). Less research has been conducted with sulfoxaflor, but the pesticide can affect the expression of proteins responsible for neurotransmitter transport and removal of neurotransmitters like glutamate (Shi et al. 2022), which is linked to memory consolidation in bee central nervous system neuroanatomy (Locatelli et al. 2005; Müßig et al. 2010).

Despite the similar mode of actions between neonicotinoids and sulfoxaflor, we had originally hypothesised that we would not observe a negative impact of sulfoxaflor on bee memory at optimal temperatures, based on the results of previous research (Siviter et al. 2019; Vaughan et al. 2022). Siviter et al. (2019) found no impact of a low, field‐realistic concentration of sulfoxaflor (250 μg dm^−3^) on bumblebee olfactory learning and memory in a traditional Proboscis Extension Reflex protocol (PER), nor spatial‐working memory in a free‐flying radial‐arm‐maze. However, the dose used by Siviter et al. (2019) is much lower than the one used in the present experiment, which likely explains the contrasting results. Vaughan et al. (2022) exposed bumblebees to both sulfoxaflor and a trypanosome parasite and found no evidence of an impact on bumblebee olfactory learning. In the present experiment, we used a dose that was identical to Vaughan et al. (2022). The contrasting results are likely caused by variation in methodologies—Vaughan et al. (2022) focused on bumblebee olfactory acquisition and utilised harnessed bumblebees in a traditional PER protocol, i.e., the bees were trained to olfactory stimuli under duress which can decrease motivation (Muth et al. 2018). We used a free‐moving experimental design, in which we trained bumblebees to learn a colour association by allowing them to approach the stimuli before a response was measured. Furthermore, in this work, the bumblebees were tasked with differentiating blue and purple, which is a relatively challenging task due to the low chromatic distance between the colours on the bee vision spectrum (Gumbert 2000; Muth et al. 2018). Thus, sulfoxaflor induced impairments in cognition may be specific to memory tasks requiring fine sensory discrimination. Future experiments should assess the impact of sulfoxaflor exposure on olfactory conditioning in a FMPER experimental design and across different sulfoxaflor doses. Regardless, these results are the first demonstrate that this insecticide can impair bumblebee memory retention, a key determinant of foraging success (Gegear et al. 2021).

We found no evidence of a temperature‐sensitive effect on bumblebee memory, contrasting previous findings (Gérard, Amiri, et al. 2022). Previous studies have shown that exposure to elevated temperatures (32°C) can impair bumblebee colour learning and memory (Gérard, Amiri, et al. 2022). Gérard, Amiri, et al. (2022), Gérard, Cariou, et al. (2022) trained bumblebees (*B. terrestris*) to learn a colour association at either 25°C or 32°C and found that bees exposed to the higher temperature acquired the association more slowly. Bees tested an hour later (either 2 h 40 min or 4 h 40 min of temperature exposure) were less likely to remember the correct colour association. Our temperature exposures (1 h) were shorter than that in the study by Gérard, Amiri, et al. (2022), Gérard, Cariou, et al. (2022), and we trained and tested bees under an optimal temperature of 26°C (Kevan et al. 2024). Had we observed an effect of temperature on bee memory, this exposure regimen would have allowed us to isolate any effects on learning, as bees were trained under optimal conditions. Furthermore, our selected temperature exposure length of 1 h reflects the length of a typical foraging bout (Westphal et al. 2006), whereas Gérard, Amiri, et al. (2022), Gérard, Cariou, et al. (2022) exposed bees for several hours, which is more reflective of multiple foraging bouts occurring in close succession. These results add to a growing body of evidence demonstrating the context specific relationship between temperature and exposure length. For example, at a colony level, both positive and negative consequences have been documented for bumblebee colonies exposed to extreme heat events (Gérard, Cariou, et al. 2022; Nebauer et al. 2024; Sepúlveda et al. 2024). Similarly, despite our results, exposure to elevated temperatures can influence bee foraging behaviour under different exposure regimes (Hemberger et al. 2023; Kenna et al. 2021, 2023; Kwon and Saeed 2003; Nooten et al. 2024). Future studies should focus on incorporating pollinator natural history to better understand the relationship between environmental temperature and pollinators’ temperature exposure. Specifically, quantifying the relationship between environmental temperature, nest temperature and bumblebee foraging activity is essential for understanding how temperature influences bumblebee colony performance (Gérard, Cariou, et al. 2022). These data will enable researchers to conduct more field‐realistic laboratory assessments that consider the relationship between temperature severity, time and behaviour.

We had predicted that co‐exposure to sulfoxaflor and temperature extremes would result in a synergistic, negative impact on bumblebee memory, but we found no evidence to support this hypothesis. Pesticide toxicity and the mechanisms responsible for the metabolic uptake of pesticides are known to be temperature‐dependent, and synergistic effects at both low and high‐temperature extremes have been demonstrated in mayflies ( *Isonychia bicolor* ), psyllids ( *Diaphorina citri* ) and damselfly larvae (*Ischnura elegans*) (Boina et al. 2009; Camp and Buchwalter 2016; Verheyen and Stoks 2023). Pesticide exposure can trigger energy‐intensive detoxification processes (Shi et al. 2022), while exposure to extreme heat can promote anaerobic metabolism in bees (Kuo et al. 2023), and lower temperatures can decrease the metabolic rate of bees due to their ectothermic physiology. As such, we would expect a larger disturbance in energy metabolism at temperature extremes, leading to synergistic effects between sulfoxaflor exposure and temperature. How this translates to sub‐lethal impacts is less clear, but previous studies have found that co‐exposure to elevated winter temperatures and sulfoxaflor resulted in a 70% decrease in longevity in solitary bees ( *Osmia cornuta* ) (Albacete et al. 2023). The same study also found negative impacts on solitary bee phototactic response behaviour and feeding behaviour. In contrast, Kenna et al. (2023), found no significant interaction between sulfoxaflor and temperature exposure in relation to bumblebee flight and feeding behaviour, although they did find negative synergistic interactions when bees were exposed to the neonicotinoid imidacloprid (Kenna et al. 2023). Likewise, co‐exposure to the commercial fungicide Pristine (active ingredients: boscalid, pyraclostrobin) and elevated temperatures resulted in synergistic, negative impacts on honeybee ( *Apis mellifera* ) homing success (DesJardins et al. 2024). In contrast, heatwave like events can increase drone production in microcolonies of bumblebees (Sepúlveda et al. 2024) and result in positive interactions with some insecticides under laboratory conditions (Nebauer et al. 2024). Future studies are required to determine whether specific agrochemicals (e.g., neonicotinoids, azole fungicides or pyrethroid) are more likely to induce synergic interactions with temperature than others, and if so, determining the molecular drivers of these interactions is vital for mitigating potential harm (Kenna et al. 2023; Siviter, Bailes, et al. 2021).

Within the Anthropocene, pollinators will be simultaneously exposed to pesticides and extreme temperatures when foraging (Ghisbain et al. 2024; Janousek et al. 2023; Nicholson et al. 2024). We found no evidence to suggest that temperature impaired bumblebee memory in this experimental setup, although exposure to the insecticide sulfoxaflor impaired bumblebee memory at field realistic concentrations. Legislation that either (i) restricts the use of sulfoxaflor entirely or (ii) limits use to non‐flowering crops will reduce the impact of this insecticide on vital pollinators (Linguadoca et al. 2021; Siviter, Brown, and Leadbeater 2018; Siviter and Muth 2020). Future studies are required to determine the relationship between sulfoxaflor and temperature severity in relation to bee cognition. More broadly, in a warming world, quantifying how environmental temperature interacts with other anthropogenic stressors is essential for predicting and mitigating the unwanted consequences of anthropogenic change (Johnson et al. 2023; Shahmohamadloo et al. 2024).

## Author Contributions

**Rinoa Hicks:** conceptualization (equal), formal analysis (lead), investigation (lead), methodology (lead), visualization (lead), writing – original draft (lead). **Szymon Szymański:** visualization (supporting), writing – review and editing (lead). **Elena Couper Coombs:** investigation (supporting). **Harry Siviter:** conceptualization (equal), formal analysis (supporting), methodology (supporting), supervision (lead), writing – original draft (supporting), writing – review and editing (supporting).

## Conflicts of Interest

The authors declare no conflicts of interest.

## Supporting information


**Data S1:** ece372073‐sup‐0001‐Supinfo.docx.

## Data Availability

All data are available online at https://osf.io/ypz39/.
