# Supplementary material for: Sulfoxaflor Impairs Bumblebee Memory Across Moderate and Extreme Temperatures
Source: Ecol Evol. 2025 Sep 5;15(9):e72073. doi: 10.1002/ece3.72073 (PMC12412415; doi:10.1002/ece3.72073)
Supplement: Supplementary file 1 — Data S1: ece372073‐sup‐0001‐Supinfo.docx. [file ECE3-15-e72073-s001.docx]

**Sulfoxaflor impairs bumblebee memory across moderate and extreme temperatures**

Rinoa Hicks*, Szymon Szymański*, Elena Couper-Coombs, Harry Siviter

School of Biological Sciences, University of Bristol, 24 Tyndall Avenue, Bristol, BS8 1TQ, UK

* *Rinoa Hicks and Szymon Szymański should be considered as joint first authors*

*Corresponding author:

Szymon Szymanski

School of Biological Sciences,

University of Bristol,

24 Tyndall Avenue,

Bristol, BS8 1TQ, UK,

[szymon.szymanski@bristol.ac.uk](mailto:szymon.szymanski@bristol.ac.uk)

**Supplementary Material**

| **Table S1.** Table of candidate models used for each analysis (top 8 presented). Treatment (control or sulfoxaflor), temperature (12°C - 36°C in 3°C increments), rewarding colour (colour of CS+), and bee size (intertegular distance). | **AICc** | **ΔAICc** | **weight** |
| --- | --- | --- | --- |
|  |  |  |  |
| **Treatment** | **586.70** | **0.00** | **0.29** |
| **Treatment + Temperature** | **587.90** | **1.23** | **0.16** |
| **Treatment + Rewarding Colour** | **588.00** | **1.38** | **0.15** |
| **Treatment + Bee Size** | **588.10** | **1.43** | **0.14** |
| Treatment + Temperature + Rewarding Colour | 589.30 | 2.60 | 0.08 |
| Treatment + Rewarding Colour + Bee Size | 589.50 | 2.81 | 0.07 |
| Treatment + Temperature + Rewarding Colour + Bee Size | 590.80 | 4.10 | 0.04 |
| Treatment:Temperature | 591.10 | 4.43 | 0.03 |

| **Table S2.** Parameter estimates and 95% confidence intervals derived from model averaging across the confidence set of models. Treatment (control or sulfoxaflor), temperature (12°C - 36°C in 3°C increments), rewarding colour (colour of CS+), and bee size (intertegular distance). | | | | |
| --- | --- | --- | --- | --- |
|  | **Estimate** | **Std. Error** | **Lower CI** | **Upper CI** |
| (Intercept) | 0.13 | 0.89 | -1.62 | 1.89 |
| **Treatment** | **-0.74** | **0.20** | **-1.14** | **-0.34** |
| Temperature | 0.00 | 0.01 | -0.013 | 0.018 |
| Rewarding Colour | -0.03 | 0.12 | -0.27 | 0.20 |
| Bee Size | 0.05 | 0.16 | -0.27 | 0.37 |
